# Comprehensive analysis of new prognostic signature based on ferroptosis-related genes in clear cell renal cell carcinoma

**DOI:** 10.18632/aging.203390

**Published:** 2021-08-09

**Authors:** Bin Zheng, Zhihong Niu, Shubin Si, Guiting Zhao, Jianwei Wang, Zhongshun Yao, Fajuan Cheng, Wei He

**Affiliations:** 1 Cheeloo College of Medicine, Shandong University, Jinan, Shandong, China; 2 Department of Urology, Shandong Provincial Hospital Affiliated to Shandong First Medical University, Jinan, Shandong, China; 3 Department of Urology, Shandong Provincial Hospital Affiliated to Shandong University, Jinan, Shandong, China; 4 Department of Urology, People's Hospital of Yiyuan County, Zibo, Shandong, China; 5 Department of Urology, Shandong Provincial ENT Hospital Affiliated to Shandong University, Jinan, Shandong, China; 6 Department of Nephrology, Shandong Provincial Hospital Affiliated to Shandong University, Jinan, Shandong, China; 7 Department of Nephrology, Shandong Provincial Hospital, Cheeloo College of Medicine, Shandong University, Jinan, Shandong, China

**Keywords:** clear cell renal cell carcinoma, ferroptosis, prognostic signature, overall survival, immune status

## Abstract

Clear cell renal cell carcinoma (ccRCC) is an aggressive tumor and the most common subtype of RCC. Ferroptosis is a novel form of regulated cell death, and ferroptosis-related genes (FRGs) have been associated with the prognosis of patients with certain cancers. However, the detailed prognostic correlation between FRGs and ccRCC has not yet been elucidated. To address this, the current study used The Cancer Genome Atlas (TCGA) dataset to explore 64 FRGs and determine their prognostic value in ccRCC. Results showed that 52 out of the 64 genes displayed significantly different expression levels in tumor tissue, and 35 out of the 52 differentially expressed genes (DEGs) were associated with overall survival. Subsequently, a four-gene prognostic signature (*CD44*, *DPP4*, *NCOA4* and *SLC7A11*) was constructed and could successfully distinguish ccRCC patients with different prognosis in TCGA train and test sets. Furthermore, clinical ccRCC samples from our medical center were used to verify the application value of the new prognostic signature through immunohistochemistry and quantitative real-time polymerase chain reaction (qRT-PCR). Biological functional analysis implied that immune-related functions and pathways were enriched in the TCGA cohort and the immune status scores were significantly different between high- and low-risk sets. These results suggest that the four ferroptosis-related regulatory genes can act as reliable prognostic biomarkers of ccRCC, and might be exploited as potential targets of therapeutic strategies.

## INTRODUCTION

Kidney or renal cancer is the 6 most common malignant cancer in males and the 8 most common in females. There will be 73,820 patients of renal cancer diagnosed in the United States, of which 14,770 patients succumbed from the disease [1]. Among the various histological types of kidney cancer, RCC is the most common type, accounting for about 85% of all cases [2]. RCCs arise from nephrons, but there are distinct histological subtypes of RCC that differ both in biology and survival outcomes. Clear cell RCC (ccRCC) is the most common subtype (70%-80% of all RCC cases) and is also one of the most aggressive subtypes [3]. Numerous treatments for ccRCC are available, including radical nephrectomy, nephron-sparing surgery, and immunotherapy, etc., but the overall prognosis has remained limited and immune-related adverse events remain to be improved [4–6]. Therefore, this calls for exploration and construction of potential prognostic models with the overarching goal of providing ccRCC patients with optimal treatments.

Previous studies confirmed that ccRCC is strongly associated with alterations in the von Hippel-Lindau (*VHL*) gene [7, 8]. Furthermore, several miRNAs (such as miR-99a, miR-106a, and miR203, etc.) and pathways (such as PI3K/AKT/mTOR, Wnt-β, and Hippo) modulate the process of ccRCC [9, 10]. In recent years, several studies have reported that ferroptosis may participate in a ccRCC-associated mechanism [11].

Ferroptosis was first introduced in 2012 by Dixon et al. [12]. As a ROS- and iron-dependent form of regulated cell death (RCD), ferroptosis is distinct from other RCDs (such as apoptosis, necroptosis, and autophagy) in both morphological changes and biochemical processes [13]. Studies have proven that diverse molecules, including *GPX4, SLC7A11* and *VDAC2/3*, regulate ferroptosis through affecting iron metabolism and lipid peroxidation [14–16]. Furthermore, recent data demonstrated that FRGs are closely associated with the prognosis of patients with hepatocellular carcinoma (HCC) [17]. However, it has not yet been elucidated whether FRGs are also associated with the prognosis of ccRCC patients.

Therefore, this study examined the expression pattern of 64 FRGs in ccRCC patients using data retrieved from TCGA database. Moreover, new risk stratification models were constructed, followed by validation of the prognostic value of the model using TCGA test cohort and clinical samples obtained from our medical center (the Shandong Provincial Hospital affiliated to Shandong First University). Finally, functional analysis was conducted to elucidate the potential mechanisms in ccRCC.

## RESULTS

### The expression pattern and correlation of ferroptosis genes in ccRCC

**Figure 1:**
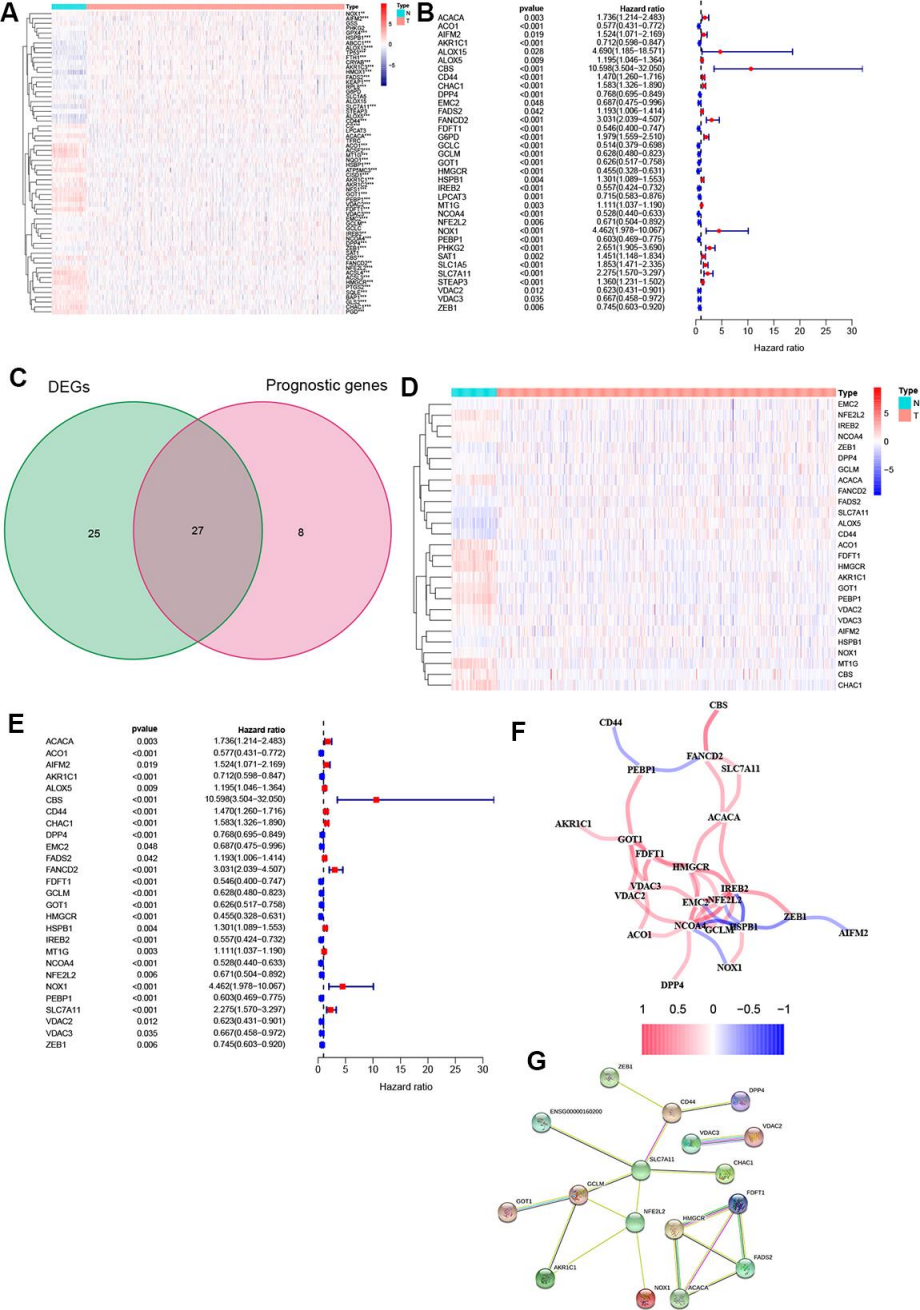
**Identification of FRGs.** (**A**) 52 genes showed significant differences in expression in ccRCC tissue. (**B**) 35 of the DEGs were associated with OS in univariate Cox analysis. (**C**) Venn plot to identify prognostic DEGs. (**D**) 27 overlapping genes show significantly different levels of expression in ccRCC tissues. (**E**) Forest plot displaying result of univariate Cox analysis between prognostic DEGS and OS. (**F**) The correlation network of prognostic DEGs. (**G**) The PPI network from the STRING database. *, *P* < 0.05; **, *P* < 0.01; ***, *P* < 0.001.

A heatmap was generated to analyze the expression pattern of FRGs in ccRCC. Most of the genes (52/64, 81.3%) showed a significantly different expression level in the ccRCC tissues compared to the normal tissues (Figure 1A). Furthermore, univariate Cox analysis revealed that 35 out of the 52 DEGs were significantly associated with OS (Figure 1B). Next, a Venn diagram was constructed to screen out the prognostic ferroptosis-related DEGs, with results showing that 27 DEGs were associated with prognosis (Figure 1C). Finally, the 27 prognostic FRGs were preserved (*p* < 0.05, Figure 1D, 1E).

Moreover, correlation analysis was used to investigate the interactions among all selected genes, with results suggesting that most of the FRGs had a positive correlation (Figure 1F). The PPI network indicated that *SLC7A11*, *GCLM,* and *NFE2L2* were the hub genes (Figure 1G).

### Construction of prognostic signature in the TCGA cohort

**Figure 2:**
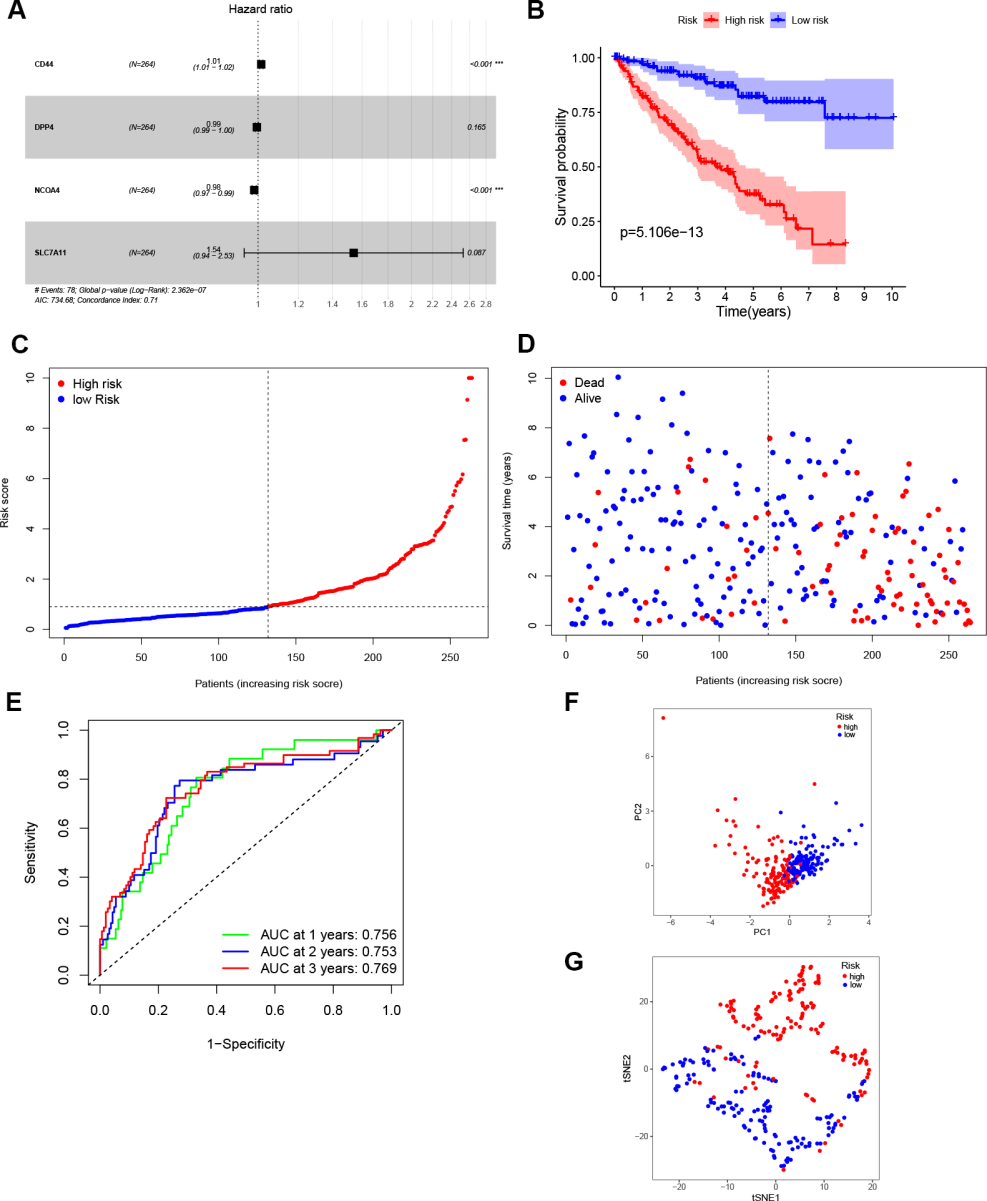
**Construction of the prognostic model.** (**A**) Construction of multivariate Cox analysis prognostic model. (**B**) K-M curve for the OS of ccRCC patients in high- and low-risk sets in the train set. (**C**, **D**) Distribution of risk scores and corresponding OS status in the train set. (**E**) ROC curve of the prognostic signature in the train set. (**F**, **G**) PCA and t-SNE analyses of the TCGA train set.

The entire TCGA cohort was randomly divided into train set (*n* = 264) and test set (*n* = 261) using the “caret” package. The 27 DEGs mentioned above were then measured as predictive genes and exposed to LASSO analysis. From the results, six FRGs were screened out based on the optimal value of λ (Supplementary Figure 1A, 1B). Subsequently, multivariate Cox analysis was performed, and a four genes prognostic model was finally constructed (Figure 2A). The risk score was then determined using the coefficients and expression level of each gene: risk score = (0.015) × *CD44* + (-0.005) × *DPP4* + (-0.017) × *NCOA4* + (0.432) × *SLC7A11*. According to the median risk score, patients in the train set were grouped into high- and low risk sets, and followed by comparison of the OS using the K-M curve. Results showed that ccRCC patients in the high-risk group had a poorer OS compared to patients in the low-risk group (Figure 2B). The distribution of the four FRGs signature based on risk scores is also displayed in Figure 2C, 2D, which was consistent with results of the K-M curve. Furthermore, time-dependent ROC analysis demonstrated that the prognostic signature had an advanced predictive performance for OS, with AUC values equal to 0.756, 0.753 and 0.769 at one, two and three years, respectively (Figure 2E). Finally, PCA and t-SNE analyses revealed that the two groups of patients were distributed in two different directions (Figure 2F, 2G).

### Validation of the four-gene signature

**Figure 3:**
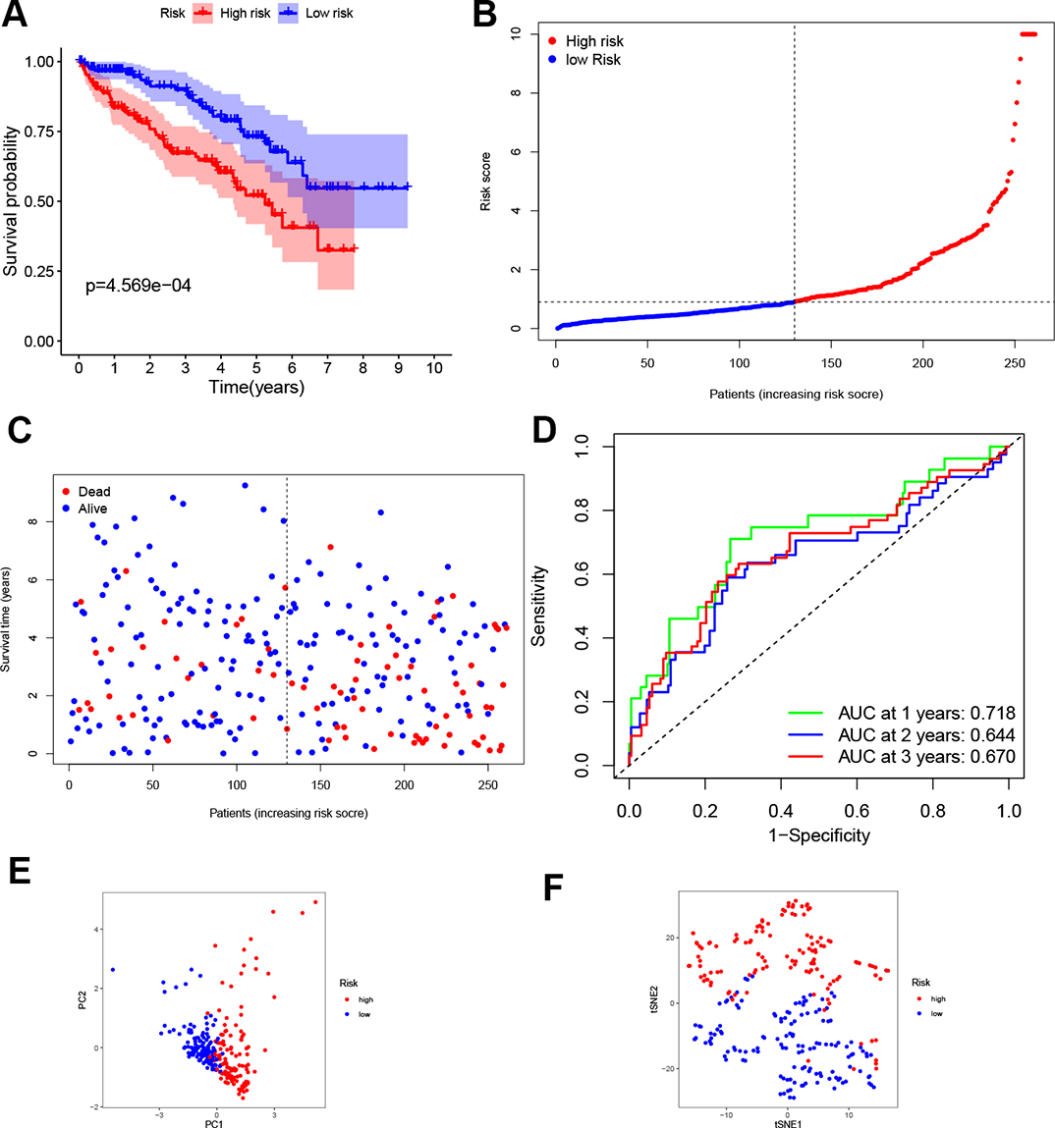
**Validation of the four-gene signature.** (**A**) K-M curve for the OS of ccRCC patients in the test set. (**B**, **C**) Distribution of the risk scores and corresponding OS status in test set. (**D**) ROC curve of the prognostic signature in test set. (**E**, **F**) PCA and t-SNE analyses of the TCGA test set.

According to the median risk score from train group, patients in test group were also categorized into high- and low-risk. Results showed that the survival outcome of high-risk patients was worse than that of low-risk patients, which was consistent with results of the train set (Figure 3A). The distribution of risk scores confirms that patients with high-risk scores have poorer survival outcomes compared to patients with low-risk scores (Figure 3B, 3C). The ROC curve demonstrated that the four-gene signature had a preferable predictive capacity for OS, with AUC values of 0.718, 0.644 and 0.670 at one, two and three years, respectively (Figure 3D). In addition, PCA and t-SNE analyses confirmed that the two groups of patients with ccRCC were dispensed in two directions (Figure 3E, 3F).

**Figure 4:**
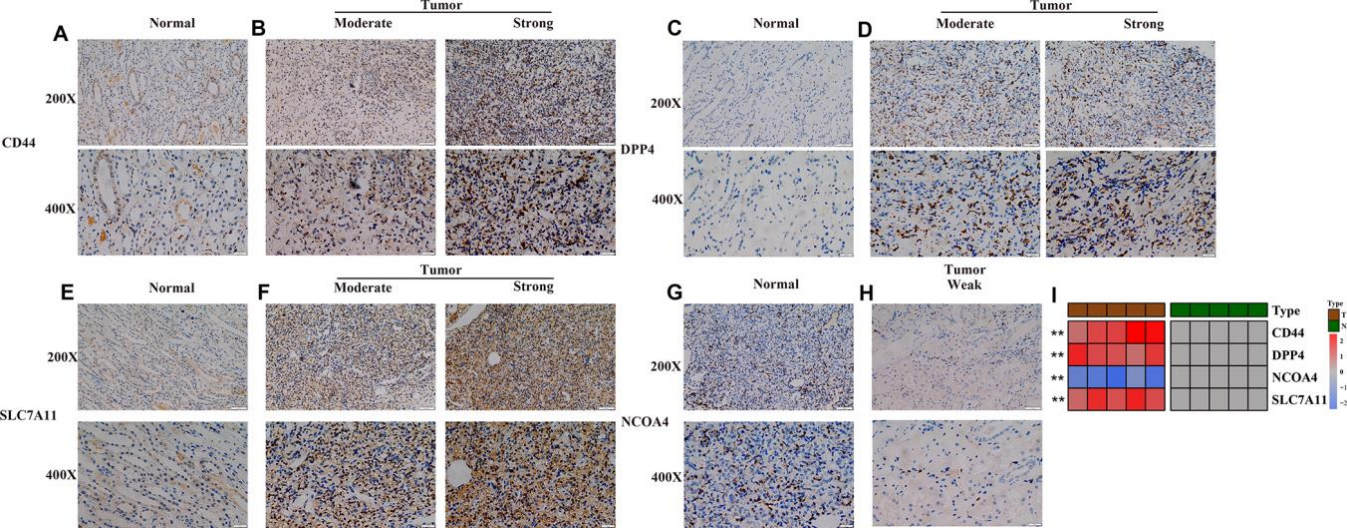
**Experimental verification of four genes in the prognostic signature.** (**A**–**H**) Immunohistochemical images of expression of the four proteins from the prognostic signature in non-tumor tissue samples (**A**, **C**, **E**, **G**) and tumor samples (**B**, **D**, **F**, **H**). (**I**) mRNA expression levels of 4 ferroptosis-related genes were evaluated using qRT–PCR in ccRCC samples and normal samples. *, *P* < 0.05; **, *P* < 0.01; ***, *P* < 0.001.

Furthermore, the robustness of the four-gene model was verified for its clinical application using KIRC samples obtained from our medical center. IHC images showed that the normal renal tissue had weak staining for *CD44*, *DPP4* and *SLC7A11* in the cytoplasm and cell membrane, while *NCOA4* displayed the opposite trend in normal tissues (Figure 4A, 4C, 4E). On the other hand, moderate and strong staining patterns for *CD44*, *DPP4*, and *SLC7A11* were observed in the cytoplasm and cell membrane of tumor tissues (Figure 4B, 4D, 4F), while *NCOA4* only exhibited weak positive staining on the cell membrane of tumor tissues (Figure 4G, 4H). These unique IHC staining patterns illustrate that the four genes can be used to predict clinical outcome and can distinguish cancerous tissue from normal tissue.

Subsequent qRT-PCR analysis also showed that the expression level of *CD44*, *DPP4* and *SLC7A11* in ccRCC samples are significantly higher than that in paired non-tumor samples (Figure 4I). In addition, the expression level of *NCOA4* in ccRCC samples is lower than that in paired non-tumor samples (*p* < 0.05, Figure 4I).

### Independent prognostic value of the constructed signature in ccRCC

**Figure 5:**
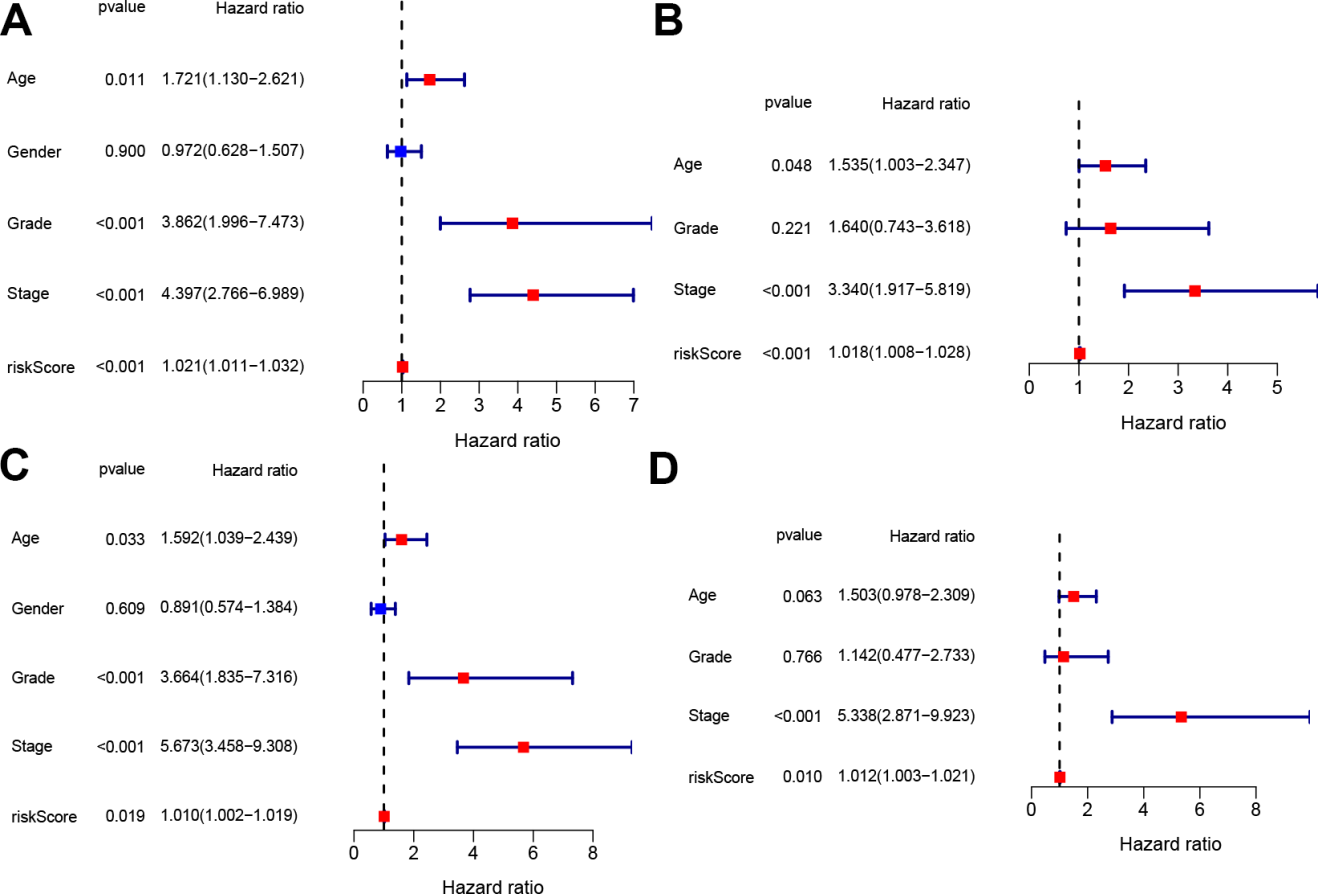
**Independent prognostic value of the constructed signature.** Univariate and multivariate Cox analyses regarding OS in the train set (**A**, **B**) and test set (**C**, **D**).

To determine whether the four-gene signature could serve as independent prognostic factor, univariate and multivariate Cox regression analyses were operated on both the train and test sets. Univariate Cox analysis indicated that age, tumor grade, tumor stage and risk score were associated with OS in both train and test sets (*p* < 0.05, Figure 5A, 5C). The variables with associated *P* value <0.1 were then enlisted into the multivariate Cox analysis, which showed that the predictors were risk score and tumor stage in both train and test sets (*p* < 0.05, Figure 5B, 5D).

### Functional analyses

**Figure 6:**
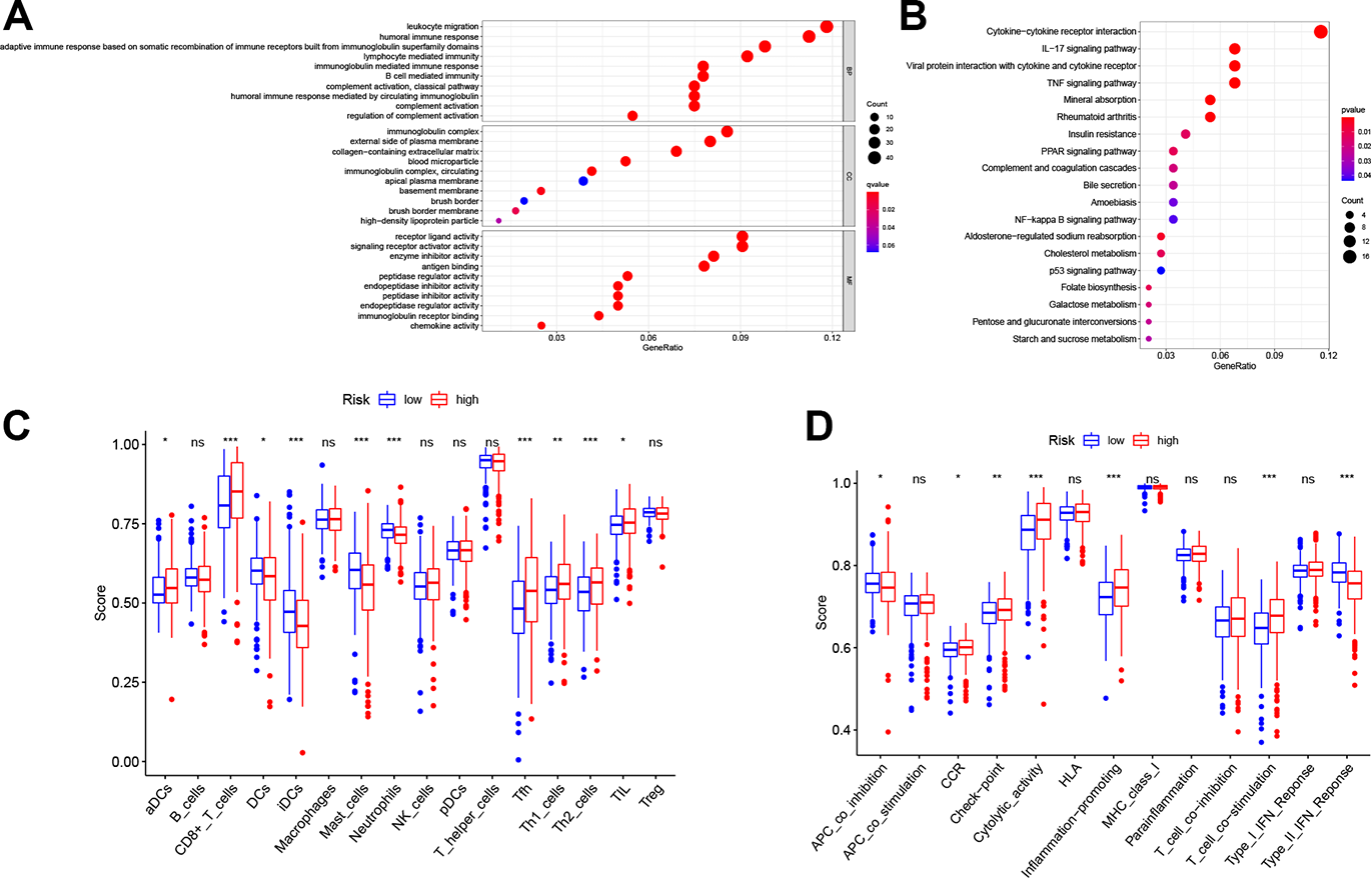
**Functional analyses in the TCGA cohort.** Representative results of the most significant GO enrichment (**A**) and KEGG pathways (**B**) in the TCGA cohort. ssGSEA scores of immune cells (**C**) and immune-related functions (**D**) between different risk groups. ns, not significant; *, *P* < 0.05; **, *P* < 0.01; ***, *P* < 0.001.

To illustrate the biological functions and pathways of genes that were differentially expressed in ccRCC tissues compared with non-cancerous tissues, GO enrichment and KEGG pathway analyses were conducted. GO analysis suggested that the DEGs were enriched in peptidase regulator activity, signaling receptor activator activity, receptor ligand activity, and plasma membrane (FDR < 0.05, Figure 6A), which is consistent with previous studies [18–21]. On the other hand, KEGG pathway analyses implied that the DEGs were enriched in the TNF signaling pathway, PPAR signaling pathway, p53 signaling pathway, folate biosynthesis, bile secretion, and cholesterol metabolism (Figure 6B), which is congruent with preceding studies [22–27]. However, the DEGs were also enriched in several immune functions and immune-associated pathways, such as cytokine-cytokine receptor interaction, lymphocyte mediated immunity, and complement activation, complement and coagulation cascades (FDR < 0.05, Figure 6A, 6B).

Considering the association between different DEGs and immune status, we further explored the interaction between risk score and immune status in the TCGA cohort. Major immune cells and functions were quantified with enrichment scores using ssGSEA. Results indicated that the scores of antigen-presenting cells (APC) and functions in the high-risk group, including DCs, iDCs and APC co-inhibition, were higher than in the low-risk group (*p* < 0.05, Figure 6C, 6D). However, the score of aDCs was lower in the high-risk group. Notably, cellular immunity, especially T cell-related immune cells and functions, including CD8^+^ T cell, Th1 cell, Th2 cell, Tfh cell and T cell co-stimulation, had higher scores in the high-risk group (*p* < 0.05, Figure 6C, 6D). Furthermore, consistent with the KEGG analysis, quantification of cytokine-cytokine receptor (CCR) interaction produced higher scores in the high-risk set (*p* < 0.05, Figure 6D). In addition, tumor infiltrating lymphocytes (TIL), the function of checkpoint regulators, and the ability of inflammation-promoting molecules displayed elevated scores in the high-risk group (*p* < 0.05, Figure 6C, 6D).

Finally, we performed co-expression analysis between four ferroptosis genes through Person’s correlation analysis. As shown in Supplementary Figure 2, based on the optimal correlation, we selected *NCOA4* and *SLC7A11* as the hub genes. Then, GSEA analysis was conducted to explore the underlying mechanisms based on the TCGA data. Notably, the results showed that overexpression of *NCOA4* was associated with renal cell carcinoma, the JAK-STAT pathway, Toll-like pathway and the NOD-like receptor pathway (|NES|> 1, *p* < 0.05, FDR < 0.25, Supplementary Figure 3A–3D). In addition, overexpressed SLC7A11 enriched in cell cycle and NOD-like receptor pathway as well (|NES|> 1, *p* < 0.05, FDR < 0.25, Supplementary Figure 3E, 3F).

## DISCUSSION

Several recent studies have recently confirmed that ferroptosis, a novel recognized form of RCD, occurs due to accumulation of lethal lipid peroxidation. Morphologically, ferroptosis is characterized by small mitochondria, reduced mitochondria crista, and ruptured outer mitochondrial membrane [28]. Emerging evidence has indicated that several compounds, such as sulfasalazine and sorafenib induce ferroptosis in cancer cells [29]. Many studies have also reported that dysregulated ferroptosis may affect multiple pathological processes, such as cancer cell death, renal failure, and T cell immunity [14, 15, 30]. Therefore, considering the significant role of ferroptosis in regulating cell death, the exact role of this process in ccRCC should be investigated.

Here, major knowledge gaps have been identified, including evaluation of FRGs to assess prognostic value as well as the potential mechanisms in ccRCC. Firstly, the expression pattern of 64 ferroptosis-related regulator genes in ccRCC was explored, with results showing that 52 of these regulators were aberrantly expressed in ccRCC. Furthermore, 35 of the 52 DEGs were associated with the prognosis of ccRCC patients. Next, 27 prognostic ferroptosis-related DEGs were screened out by Venn diagram, and a prognostic model with four genes (*CD44*, *DPP4*, *NCOA4*, and *SLC7A11*) was developed via LASSO and multivariate Cox regression analyses. With the median risk score, patients were then grouped into low- and high-risk groups. The K-M curve and ROC curve indicated that this four-gene signature had a good ability to predict prognosis. In addition, multivariate Cox analysis showed that the risk score was an independent prognostic factor. Besides, the prognostic signature was validated using the test set and in KIRC samples from our hospital, with results further suggesting the prognostic value of the four-gene signature in clinical application. Finally, GO and KEGG enrichment analyses were performed in the TCGA cohort. In addition to the ferroptosis-related functions and pathways, several tumor immune-related functions and pathways were enriched. Moreover, ssGSEA results showed that the APCs scores and cellular immunity scores, especially those of T cell-related immune cells, were also higher in the high-risk group than in the low-risk group. It has been proposed that ferroptotic cells may release diverse ‘find me’ signaling molecules, which could attract APCs or other immune cells to the location of ferroptotically dying cells [11]. Furthermore, T cell immunity, especially CD8^+^ T cells could promote ferroptosis-specific lipid peroxidation in tumor cells, thereby contributing to the antitumor efficacy of immunotherapy [31]. Therefore, the prognostic models established in this study have the potential to evaluate the prognosis of ccRCC patients. Meanwhile, the four selected ferroptosis-related regulatory genes can be exploited as potential targets of therapeutic strategies and the relevant mechanisms should be explored further.

Specifically, the four genes in the prognostic signature perform distinct functions in the process of ferroptosis. For example, *NCOA4* plays important role in regulating iron metabolism. Several studies reported that knocking down these genes suppressed erastin-induced ferroptosis and/or amino acid/cystine deprivation-induced ferroptosis [32, 33]. In addition, *DPP4* inhibited erastin-induced ferroptosis in colorectal cancer after its activity was blocked by *p53*. However, in the absence of *p53*, *DPP4* combines with *NOX1* to form the *NOX-DPP4* complex, thereby leading to plasma membrane lipid peroxidation and ferroptosis [21, 34]. *SLC7A11* participates in many ferroptosis-related signaling pathways. Studies have reported that *BAP1* could promote ferroptosis by blocking the expression of *SLC7A11*. Moreover, *SLC7A11* could induce ferroptosis when the African-restricted polymorphism S47 in the *p53* (*p53^3KR^*) gene was mutated [26, 35]. *CD44*, a marker of cancer stem cells, has also been shown to regulate iron homeostasis and modulate iron entry into tumor cells [36]. In particular, *CD44v*, the isoform of *CD44*, could stabilize protein xCT and hence stimulate glutathione synthesis, which then further undermined ROS-induced stress signaling, a ferroptosis hallmark [37]. However, despite these observations, there are only limited studies concerning the mechanism of ferroptosis in ccRCC. Therefore, additional experimental proof about these ferroptosis-related regulators in ccRCC is needed.

There are several limitations in this current study. Firstly, the ccRCC cohort is relatively small and clinical data of the samples is not complete. Secondly, the lack of *in vitro* experimental verification affects the rigor of this study. Although we conducted GO and KEGG analysis, the specific upstream and downstream target molecules as well as the specific regulatory mechanisms still need to be verified. Therefore, future studies should include the prospective data of ccRCC patients and *in vitro* experimental verification in order to strengthen the findings of this study. Overall, this research is far from being completed and most likely this process will continue in the next years. Given the limited knowledge of ferroptosis-related genes, it is of critical urgency to interrogate detailed mechanism and novel targets for future clinical application.

Collectively, the finding of this study illustrated a dysregulated expression profile of FRGs between ccRCC and normal samples. Many clinicopathological features, including patients’ age, tumor grade and stage could act as independent prognostic factors, which illustrated the pivotal role of these genes in the development of ccRCC. Finally, a four-gene prognostic model was constructed to determine various prognoses of ccRCC patients, and the model implied that *CD44*, *DPP4*, *NCOA4*, and *SLC7A11* may serve as potential molecular biomarkers of ccRCC.

## MATERIALS AND METHODS

### Data collection

Expression data and clinical information of 539 ccRCC and 72 normal patients were obtained from the TCGA database (https://portal.gdc.cancer.gov/). Expression data were normalized by “limma” package. After performing a comprehensive literature review, 64 FRGs were identified [13, 19, 38–41] (Supplementary Table 1).

### Selection of intersected genes between differential expression- and prognostic ferroptosis-related genes

DEGs in paired tumor and non-tumor tissues were assessed by “limma” package. Univariate Cox analysis of overall survival (OS) was then conducted to obtain prognostic genes. Finally, intersected FRGs were screen out using the “venn” R package. The differential expression patterns, co-expression correlation analysis, and prognostic values were visualized by “survival”, “igraph” and “pheatmap” R package.

### Construction and validation of the prognostic signatures

The TCGA cohort was randomly divided into two groups (train and test groups) by using the “caret” package. Least absolute shrinkage and selection operator (LASSO) Cox regression analysis was conducted in train set with the "glmnet" R package. Multivariate cox analysis was performed to build prognostic models. The median value of risk scores was calculated to stratify ccRCC patients into high-risk and low-risk sets by the following formula: $\text{Risk}\;\text{score}=\sum_{1}^{n}Coef_{n}\times x_{n}$ (where Coef_n_ is the coefficient and x_n_ is the expression level of each intersected gene). Next, a Kaplan-Meier (K–M) curve was used to analyze the OS in train and test groups, respectively. Receiver operating characteristic (ROC) analysis was also executed to assess the prediction efficiency of prognosis. Based on the expression profile of four FRGs, PCA and t-SNE analysis were performed to demonstrate the expression patterns of various FRGs in diverse groups. Finally, IHC pattern was certified by utilizing KIRC clinical samples from our hospital. Univariate and multivariate Cox analyses were utilized to investigate independent prognostic factors for ccRCC patients.

### Functional analyses

A protein–protein interaction (PPI) network was built for prognostic DEGs by the STRING database (http://string-db.org/). Gene Ontology (GO) and Kyoto Encyclopedia of Genes and Genomes (KEGG) analyses based on the DEGs were then operated by utilizing "clusterProfiler" package. In addition, the score of major immune cells and immune-related pathways were determined with single-sample gene set enrichment analysis (ssGSEA). GSEA was performed using GSEA v4.1.0 (http://www.broadinstitute.org/gsea/).

### Immunohistochemistry

Ten pairs of ccRCC and adjacent normal tissues were collected from Shandong provincial Hospital affiliated to Shandong First University during January 2021 to February 2021. The experiment was approved by the Shandong Provincial Hospital Ethics Committee (Approval number: SWYX: NO.2021-118) and written informed consents were signed by each patient before the study began. IHC was performed according to previously described standard procedures [42]. All samples were incubated with rabbit polyclonal antiCD44 (ab189524), antiNCOA4 (ab62495), antiDPP4 (ab187048) and antiSLC7A11 (ab175186) antibodies overnight at 4° C and were then washed. Two pathologists independently assessed the IHC slides.

### RNA analysis, extraction, and quantitative real-time PCR

Total RNA of frozen tissue was extracted by TRIzol reagent (Tiangen Biotech (Beijing)), and 1 μg of total RNA was reverse transcribed using the FastKing RT Kit (Tiangen Biotech (Beijing)) according to the manufacturer’s instructions. Followed by measuring with a real-time quantitative PCR system. The primers used in this study were provided by Beijing Dingguo Changsheng Company and are shown in Supplementary Table 2. The expression data was log2 transformed: log2(exp + 0.01).

### Statistical analysis

Mann-Whitney tests were utilized to measure gene expression between ccRCC and non-tumor tissues. Associated samples with incomplete clinical information were eliminated. All data analyses were conducted using the R statistical package (R version 4.0.1). A two-tailed *p* < 0.05 was considered statistically significant.

### Data availability statement

The data used to support the findings of this study are available from the corresponding author upon request.

## Supplementary Material

Supplementary Figures

Supplementary Tables
