# Supplementary material for: Comprehensive analysis of new prognostic signature based on ferroptosis-related genes in clear cell renal cell carcinoma
Source: Aging (Albany NY). 2021 Aug 9;13(15):19789–804. doi: 10.18632/aging.203390 (PMC8386570; doi:10.18632/aging.203390)
Supplement: Supplementary Figures [file aging-13-203390-s001.pdf]

SUPPLEMENTARY FIGURES

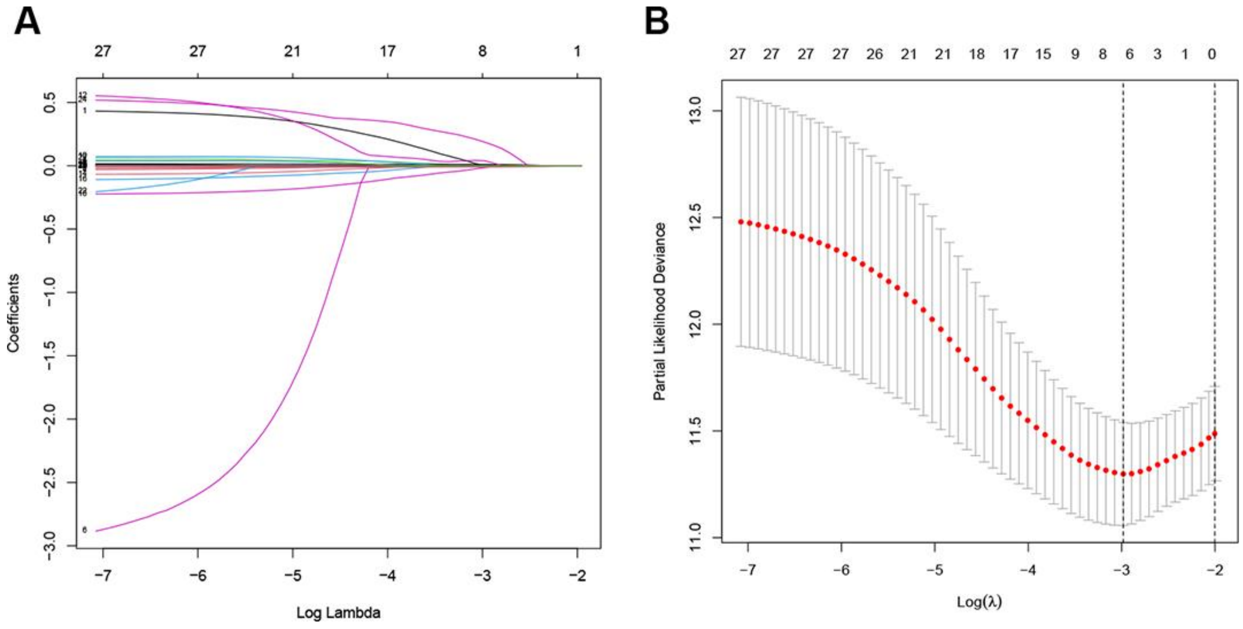

Supplementary Figure 1. LASSO regression analysis of 27 prognostic DEGs. (**A**) LASSO coefficient profiles of 27 genes. (**B**) Selection of the penalty parameter (λ).

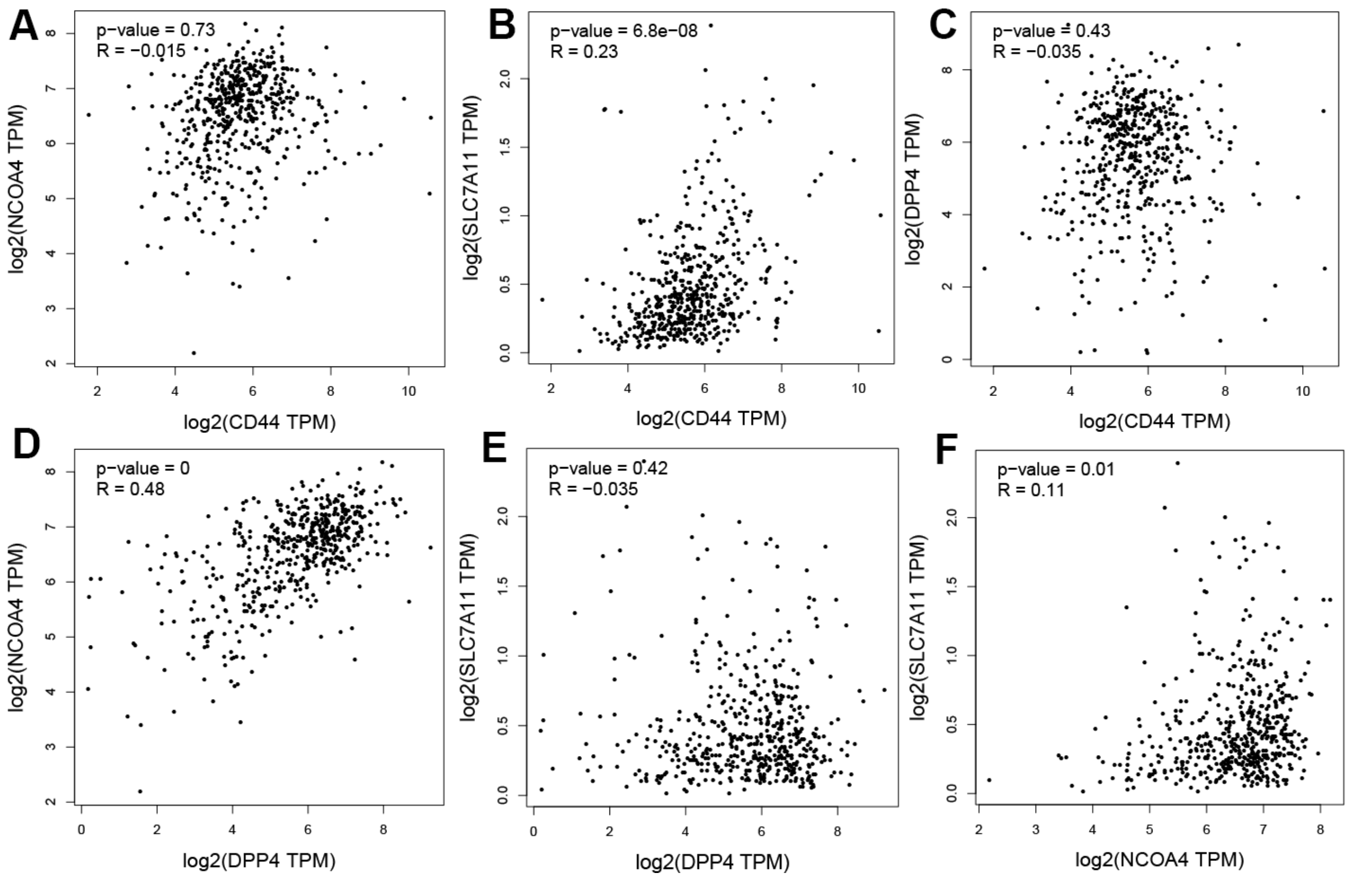

Supplementary Figure 2. (**A**–**F**) Co-expression analysis between four ferroptosis genes.

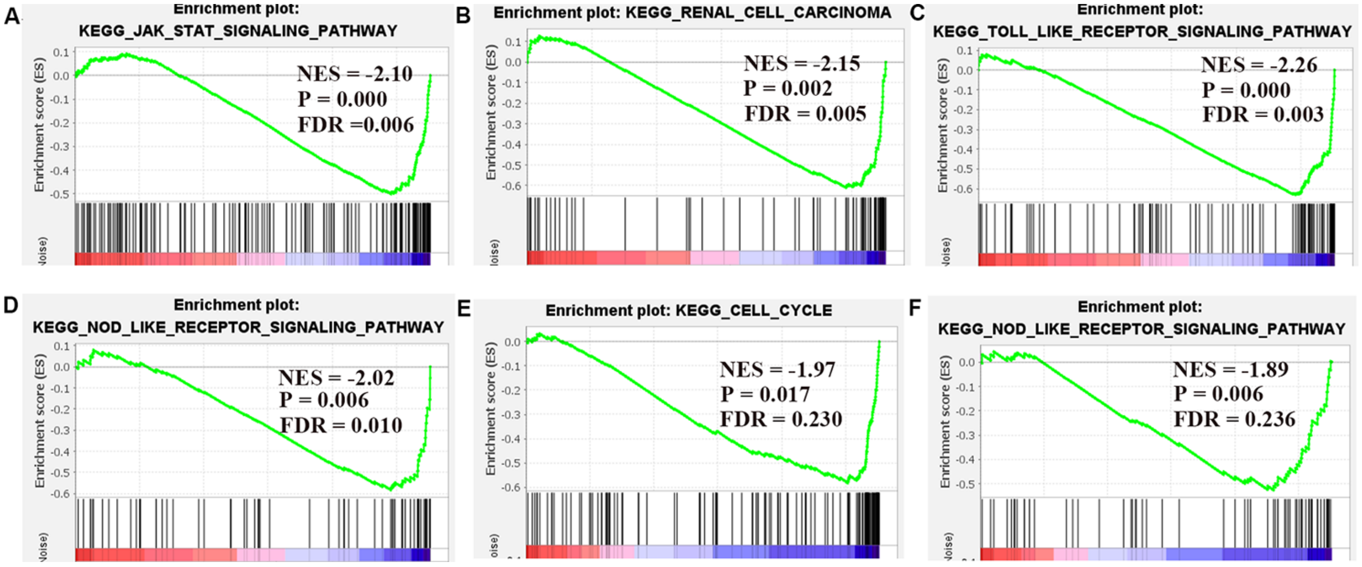

Supplementary Figure 3. (**A**–**F**) Enrichment curves for NCOA4 (**A**–**D**) and SLC7A11 (**E**, **F**) by GSEA analysis. NES, normalized enrichment score; FDR, false discovery rate.
