# Supplementary material for: Comprehensive analysis of new prognostic signature based on ferroptosis-related genes in clear cell renal cell carcinoma
Source: Aging (Albany NY). 2021 Aug 9;13(15):19789–804. doi: 10.18632/aging.203390 (PMC8386570; doi:10.18632/aging.203390)
Supplement: Supplementary Tables [file aging-13-203390-s002.pdf]

SUPPLEMENTARY TABLES

Supplementary Table 1. The list of the 64 ferroptosis-related genes from publications.

Ferroptosis-related genes	Name
ABCC1	ATP binding cassette subfamily C member 1
ACACA	Acetyl-CoA carboxylase alpha
ACO1	aconitase 1
ACSF2	acyl-CoA synthetase family member 2
ACSL3	acyl-CoA synthetase long-chain family member 3
ACSL4	acyl-CoA synthetase long-chain family member 4
AIFM2	apoptosis inducing factor mitochondria associated 2
AKR1C1	aldo-keto reductase family 1 member C1
AKR1C2	aldo-keto reductase family 1 member C2
AKR1C3	aldo-keto reductase family 1 member C3
ALOX12	arachidonate 12-lipoxygenase
ALOX15	arachidonate 15-lipoxygenase
ALOX5	arachidonate 5-lipoxygenase
ATP5MC3	ATP synthase membrane subunit c locus 3
BAP1	BRCA1 associated protein 1
CARS	cysteinyl tRNA synthetase
CBS	cystathionine beta synthase
CD44	CD44 molecule
CHAC1	ChaC glutathione- specific gamma-glutamyl cyclotransferase 1
CISD1	CDGSH iron sulfur domain 1
CRYAB	heat shock protein beta 5
CS	citrate synthase
DMT1	ferrous ion membrane transport protein DMT1
DPP4	dipeptidyl-dippeptidase-4
EMC2	ER membrane protein complex subunit 2
FADS2	fatty acid desaturase 2/acyl-CoA 6-desaturase
FANCD2	Fanconi anemia complementation group D2
FDFT1	farnesyl-diphosphate farnesyltransferase 1
FTH1	ferritin heavy chain 1
G6PD	glucose-6-phosphate dehydrogenase
GCLC	glutamate-cysteine ligase catalytic subunit
GCLM	glutamate-cysteine ligase modifier subunit
GLS2	glutaminase 2
GOT1	glutamic-oxaloacetic transaminase 1
GPX4	glutathione peroxidase 4
GSS	glutathione synthetase
HMGCR	3-hydroxy-3- methylglutaryl-CoA reductase
HMOX1	heme oxygenase 1
HSBP1	heat-shock 27-k Da protein 1
HSPB1	heat shock protein beta 1
IREB2	iron response element-binding protein 2
KEAP1	kelch-like ECH- associated protein 1
LPCAT3	lysophosphatidylcholine acyltransferase 3
MT1G	metallothionein-1G
NCOA4	nuclear receptor coactiva tor 4
NFE2L2	nuclear factor, erythroid 2 like 2

NFS1	cysteine desulfurase
NOX1	NADPH oxidase 1
NQO1	quinone oxidoreductase-1
PEBP1	phosphatidylethanolamine-binding protein 1
PGD	phosphoglycerate dehydrogenase
PHKG2	phosphorylase kinase, g2
PTGS2	prostaglandin-endoperoxide synthase 2
RPL8	ribosomal protein L8
SAT1	spermidine/spermine N1-acetyltransferase 1
SLC1A5	solute carrier family 1 member 5
SLC7A11	solute carrier family 7 member 11
SQLE	squalene monooxygenase
STEAP3	six-transmembrane epithelial antigen of prostate 3
TFRC	transferrin receptor
TP53	tumor protein 53
VDAC2	voltage dependent anion channel 2
VDAC3	voltage dependent anion channel 3
ZEB1	zinc finger E-box-binding homeobox 1

Supplementary Table 2. Primers of 4 ferroptosis-related genes used in this study.

Genes	Primers (5'-3')
DPP4-F	TTCAGAACTATTCGGTCA
DPP4-R	ATAAAGATTCCTTCCTCCT
NCOA4-F	TCAACATAGAACGCACAT
NCOA4-R	AGAACTCCACCAATAGCA
SLC7A11-F	TATTCTATGTTGCGTCTCG
SLC7A11-R	TATCATTGTCAAAGGGTGC
CD44-F	CAGCTCATACCAGCCATCCA
CD44-R	GCTTGATGACCTCGTCCCAT
Actin-F	ACACTGTGCCCATCTACG
Actin-R	TGTCACGCACGATTTCC
